# Physiology of the Soul and Instinct, as Distinguished from Materialism

**Published:** 1872-04

**Authors:** 


*Physiology of the Soul and Instinct, as distinguished from Materialism.* By Martin Paine, A.M., M.D., LL.D., etc. New York: Harper Bros, 1872.

To give an extended and just notice of this truly learned and interesting work, requires more space than our pages can afford in the present issue. The several subjects treated of are taken up in the regular order and show evidence of being treated by a master hand. The present work may be considered as an enlarged and revised edition of the work of Prof. Paine, published in 1848, on the Physiology of the Soul and Instinct. The author, although stating his ideas in a clear and concise manner, and replying to the theories of some of the modern Philosophers in his well known vigorous style, seems to us, to have too little charity for those who hold opinoins in opposition to his own. The whole subject is ably treated, and the illustrations clearly and forcibly drawn. The work cannot fail of being accorded a high position in the literature on the subject of which it treats.

